# Supplementary material for: Anti-Osteoarthritic Effects of Prunella Vulgaris and Gentiana Lutea In Vitro and In Vivo
Source: Antioxidants (Basel). 2022 Dec 26;12(1):47. doi: 10.3390/antiox12010047 (PMC9854930; doi:10.3390/antiox12010047)
Supplement: Supplementary file 1 [file antioxidants-12-00047-s001.zip › antioxidants-2098862-supplementary.pdf]

Effects of loganin on bone formation and resorption *in vitro* and *in vivo*

Chang-Gun Lee, Do-Wan Kim, Jeonghyun Kim, Laxmi Prasad Uprety, Kang-Il Oh, Shivani Singh, Jisu Yoo, Hyun-Seok Jin, Tae Hyun Choi, Eunkuk Park and Seon-Yong Jeong

Supplementary Table S1. Gene specific primers used in this study.

Gene	Primer sequence
Alpl	Forward: 5′-TCC CAC GTT TTC ACA TTC GG-3′<br>Reverse: 5′-CCC GTT ACC ATA TAG GAT AGC C-3′
Runx2	Forward: 5′-TAA AGT GAC AGT GGA CGG TCC C-3′<br>Reverse: 5′-AAT GCG CCC TAA ATC ACT GAG G-3′
Bglap	Forward: 5′-TAG TGA ACA GAC TCC GGC GCT A-3′<br>Reverse: 5′-TGT AGG CGG TCT TCA AGC CAT-3′
Ctsk	Forward: 5′-AAT ACC TCC CTC TCG ATC CTA CA-3′<br>Reverse: 5′-TGG TTC TTG ACT GGA GTA ACG TA-3′
Acp5	Forward: 5′-TGG TAT GTG CTG GCT GGA AAC-3′<br>Reverse: 5′-GCA GAG GCA TAC TTG TAC CG-3′
Mmp9	Forward: 5′-GCA GAG GCA TAC TTG TAC CG-3′<br>Reverse: 5′-TGA TGT TAT GAT GGT CCC ACT TG-3′
Gapdh	Forward: 5′-AGG TCG GTG TGA ACG GAT TTG-3′<br>Reverse: 5′-TGT AGA CCA TGT AGT TGA GGT CA-3′
Hprt	Forward: 5′-GAG GAG TCC TGT TGA TGT TGC CAG-3′<br>Reverse: 5′-GGC TGG CCT ATA GGC TCA TAG TGC-3′
